# Species richness and asynchrony maintain the stability of primary productivity against seasonal climatic variability

**DOI:** 10.3389/fpls.2022.1014049

**Published:** 2022-10-28

**Authors:** Ze Zhang, Yann Hautier, Tiejun Bao, Jie Yang, Hua Qing, Zhongling Liu, Min Wang, Taoke Li, Mei Yan, Guanglin Zhang

**Affiliations:** ^1^ Ministry of Education Key Laboratory of Ecology and Resource Use of the Mongolian Plateau, Inner Mongolia University, Hohhot, China; ^2^ Inner Mongolia Key Laboratory of Grassland Ecology, School of Ecology and Environment, Inner Mongolia University, Hohhot, China; ^3^ Ecology and Biodiversity Group, Department of Biology, Utrecht University, Padualaan, Utrecht, Netherlands

**Keywords:** climate variability, species richness, asynchrony, temperate grassland, community temporal stability

## Abstract

The stability of grassland communities informs us about the ability of grasslands to provide reliable services despite environmental fluctuations. There is large evidence that higher plant diversity and asynchrony among species stabilizes grassland primary productivity against interannual climate variability. Whether biodiversity and asynchrony among species and functional groups stabilize grassland productivity against seasonal climate variability remains unknown. Here, using 29-year monitoring of a temperate grassland, we found lower community temporal stability with higher seasonal climate variability (temperature and precipitation). This was due to a combination of processes including related species richness, species asynchrony, functional group asynchrony and dominant species stability. Among those processes, functional group asynchrony had the strongest contribution to community compensatory dynamics and community stability. Based on a long-term study spanning 29 years, our results indicate that biodiversity and compensatory dynamics a key for the stable provision of grassland function against increasing seasonal climate variability.

## Introduction

In recent decades, we have witnessed severe climate changes including warmer temperature as well as more variable precipitation patterns such as frequent floods and droughts (Kardol et al., 2010; Min et al., 2011; Orlowsky and Seneviratne, 2012; IPCC, 2013; Thornton et al., 2014; Putnam and Broecker, 2017). There is growing concern that increased climate variability may affect the structure, function and temporal stability of grassland ecosystems (Huxman et al., 2004; Vargas et al., 2012; Grant et al., 2014; Peralta et al., 2019; Liu et al., 2021). The temporal stability of grassland communities, usually measured as the invariability of community biomass among years, is key to provide reliable services including nutrient and carbon cycling as well as fodder for livestock. Understanding the influence of climatic changes, particularly seasonal variability, on grassland community temporal stability is essential since dairy and meat farmers around the world rely on stable fodder production for stable income (Lurette et al., 2013; Bengtsson et al., 2019).

Previous studies have shown that increased precipitation variability generally reduces primary productivity (Knapp et al., 2002; Gherardi and Sala, 2015; Wagg et al., 2017) and its temporal stability (Zhang et al., 2018). However, these effects may depend on the difference in environmental conditions such as climate and soil caused by differences in geographical location (Liu et al., 2019). Additionally, climate variability can alter functional trait composition, such as plant height, leaf area and leaf dry matter content (Cheng et al., 2021; Shaw et al., 2022), and reduce the species richness of grassland ecosystems (Thomas et al., 2004; Wu et al., 2012; Cleland et al., 2013). These changes in plant diversity, community composition and primary productivity in response to increased climate variability may in turn affect the temporal stability of grassland community.

Theoretical and empirical evidence suggests that grassland community temporal stability is influenced by multiple underlying mechanisms (Loreau and De Mazancourt, 2013; Ma et al., 2017).

First, dominant species often plays an important role in the community (Grime, 1998, mass-ratio hypothesis) and the temporal stability of dominant species may disproportionately contribute to the temporal stability of the whole community (Xu et al., 2015; Ma et al., 2017). Similarly, the temporal stability of functional groups with higher relative abundance may disproportionately contribute to community temporal stability (Wilsey et al., 2014; Huang et al., 2020).

Second, species asynchrony (different responses of species to environmental change) can maintain community temporal stability by altering the complementarity of the relative biomass of species in different niches (Gross et al., 2014; Hallett et al., 2014; Wilcox et al., 2017). However, biomass complementarity may be stronger among functional groups due to their higher relative biomass (Bai et al., 2004). In this case, species asynchrony may be a weaker predictor of temporal stability compared to functional group asynchrony (Díaz and Cabido, 2001; Bai et al., 2004). Therefore, considering both species and functional group asynchrony may help to improve our understanding of community temporal stability (Zhou et al., 2019).

Third, species diversity and community composition can affect community temporal stability directly by altering the temporal mean of productivity or indirectly by affecting species/functional group temporal stability and asynchrony. For example, higher plant diversity usually increases the productivity of grassland communities, leading to higher stability *via* overyielding (Hautier et al., 2015). Communities dominated by fast-growing species may show lower resistance but higher resilience, while communities dominated by slow-growing species may show the reverse (Craven et al., 2018). Thus, communities with a high diversity of fast-slow traits should buffer community response to environmental changes and be more stable (Grime et al., 2008; Hector et al., 2010; Fry et al., 2018). For resource-limited grassland ecosystems, species diversity can facilitate competition for and use of resources by dominant species, and the temporal stability of dominant species or functional group can play a stabilizing role (Bartha et al., 2014; Doležal et al., 2019). Species diversity may further improve community temporal stability by promoting species asynchrony (Loreau and De Mazancourt, 2008; Sasaki et al., 2019) and functional group asynchrony (Lehman and Tilman, 2000).

Long-term monitoring can reveal the long-term dynamic of plant communities in response to climate variability, and the relationship between community temporal stability and long-term climate variability (Bai et al., 2004; Li et al., 2015; Zhou et al., 2019). So far, previous studies have focused on the relationship between climate variability and interannual stability of community. However, climate variability is likely to be stronger on seasonal scales than on annual scales (Donat et al., 2016; Zhang et al., 2018; Zhang et al., 2022). Additionally, the response and underlying mechanisms shaping community temporal stability in response to seasonal climate variability remain unresolved (Ives and Carpenter, 2007; Smith et al., 2017). Here, we quantify the link between seasonal climate variability and grassland community temporal stability. We collected long-term monthly data on community biomass, community composition, species richness and climate of a temperate grassland between 1981 and 2011 in northern China, and analyzed the relationships of seasonal temperature and precipitation variability on seasonal community temporal stability. We hypothesize that plant community would be less stable with higher seasonal climate variability because 1) higher seasonal climate variability reduces the positive effect of species richness and species/functional group asynchrony, and 2) higher seasonal climate variability reduces the temporal stability of dominant species/functional group.

## Materials and methods

### Study site

The observation was carried out at the Inner Mongolia Grassland Ecosystem Research Station (IMGERS, 116.8°E, 43.5°N, 1179 m a.s.l.), located in a temperate grassland in the Inner Mongolia, China (**Supplementary Figure 1**). The study area has a temperate continental climate, with hot-rainy summer and cold-dry winter. During the observation period of this study (1981-2011), the mean annual temperature was 0.77°C and the mean monthly minimum temperature was -21.3°C in January, with the mean monthly maximum temperature of 19.3°C in July (**Supplementary Figure 2**). The annual precipitation was 330.7 mm and approximately 83.8% of precipitation fell in the growing season (from May to September). According to Chinese classification, the soil type is chestnut soil, and Calcis-orthic Aridisol in the US Soil Taxonomy classification, with an average bulk density of 1.29 g cm^−3^ in 0-20 cm soil layer and a pH of 7.68 (Yuan et al., 2005; Song et al., 2016).

### Experimental design

The study area is an 18-ha (600 m × 300 m) rectangular homogeneous area fenced since 1979 to prevent grazing by large animals (Li et al., 2015). It was equally separated into ten replicate blocks (60 m ×300 m) for aboveground biomass monitoring in 1981. Community aboveground biomass was surveyed in the middle of every month throughout the growing season (from May to September) of each year by clipping green parts of all vascular plants above the soil surface within a 1 m × 1 m quadrat that was randomly located within each block, over 1981–2011. Hence, community aboveground biomass was estimated for 1450 quadrats (i.e., 1 quadrat × 10 blocks per month × 5 months per year × 29 years = 1450 quadrats) excluding missing data from the years 1995 and 1996 (Ma et al., 2010). For each survey, the location of the quadrat was marked to avoid setting up quadrat at the same site. Other areas in each block that were not harvested remained undisturbed. After harvesting, all plants were sorted into species, and oven-dried at 65 °C to a constant weight, and then weighed. The relative abundance of the aboveground dry biomass of each species in the total aboveground dry biomass of the community was calculated, and the species with relative abundance > 5% was determined as the dominant species (Xu et al., 2015; Ma et al., 2017), including two perennial rhizome grasses, *Leymus chinensis* (25.5 ± 4.2%) and *Agropyron cristatum* (7.1 ± 1.6%), and two perennial bunchgrasses, *Stipa grandis* (19.1 ± 3.6%) and *Achnatherum sibiricum* (11.2 ± 2.7%). These dominant species accounted for 62.9 ± 10.1% of the total aboveground dry biomass of the community at the study site. According to different life forms, all species were further divided into three functional groups, including perennial grass (PG), perennial forbs (PF), and other plants (OP) (including shrubs, semi-shrubs, annuals and biennials). Species richness was recorded in the same plot in which aboveground biomass was measured. A total of 61 species were found during 1981 and 2011.

### Climate data

The monthly mean temperature (daily mean then averaged to the month), and monthly cumulative precipitation data were collected from the IMGERS weather station situated about 9 km from the study site. Climate variability was expressed as the inter-annual coefficients of variation (CV) of climate factors, and the calculation formula was σ/μ×100, where σ and μ were the inter-annual temporal standard deviation and mean of monthly temperature or cumulative precipitation for May, June, July, August, September and the whole growing season over the period 1981–2011.

### Statistical analysis

Data collected over a period of 29 years, from 1981 to 2011, were used in this analysis excluding missing data from the years 1995 and 1996 (Ma et al., 2010). Similar to climatic variability, the community temporal stability was calculated as μ/σ (Ma et al., 2017), where μ was the inter-annual mean monthly biomass of the community from 1981 to 2011, and σ was the standard deviation. The functional group and dominant species (*L. chinensis, A. cristatum, A. sibiricum and S. grandis*) temporal stability was calculated using the same method. A higher value of community stability means a lower inter-annual variability of community biomass (Lehman and Tilman, 2000).

Species asynchrony, which refers to the asynchronous response of species to environmental fluctuations (Loreau and De Mazancourt, 2008), was calculated as


$$1-\varphi_{x}=1-\rho^{2}/{\left(\sum_{l=1}^{T}\rho_{l}\right)}^{2}$$

where *φ_x_* was species synchrony, *ρ* ^2^ and *ρ_l_* were the variance of community biomass, and the standard deviation of biomass of species *l* in a plot with *T* species for each month (May to September) across the 29 years. Species asynchrony, fluctuates between 0 and 1, and higher values indicate stronger asynchronous changes among species in the community.

The annual change rates of monthly mean temperature and precipitation were calculated by using the slope of linear regression equation of temperature or precipitation with year. Repeated measures analysis of variance (ANOVA) was used to test the variation of species richness and community biomass in different growing seasons. Using SPSS 19.0 software package, one-way ANOVAs were employed to probe the means differences in species asynchrony, functional group asynchrony, dominant species temporal stability and community temporal stability among months within growing season, and the alpha significance level was 0.05. Simple linear regression was used to analyze the relationships between community temporal stability and species richness, species asynchrony, functional group asynchrony, dominant species temporal stability, temporal stability of PG, PF and OP, and precipitation or temperature variability. All the variables used in the analysis conformed to the normal distribution.

Using AMOS 22.0 software package, structural equation modeling (SEM) was used to assess the effects of climate variability on community temporal stability through species richness, species asynchrony, functional group asynchrony, functional group temporal stability, and dominant species temporal stability at different months of the growing season from 1981 to 2011. Based on regression weight estimation, the initial model was simplified and non-significant path and state variables were eliminated, so the final model contained only statistically significant paths that cannot be rejected (**Supplementary Table 1-6**). According to the non-significant path coefficient and the level of significance for the region weight, the analysis result of structural equation model was drawn for different months of the growing season. The stronger the level of significance for the region weight, the higher the correlation. Accuracy of the model was confirmed using a Chi-squared test, the Akaike Information Criterion (AIC) and the root-mean-square errors of approximation (RMSEA). The model has a good fit when Chi-squared test *χ* ^2^ ≥ 0,P > 0.05, lower AIC and 0 ≤ RMSEA ≤ 0.08.

## Results

### Trends in climate change

**Figure 1:**
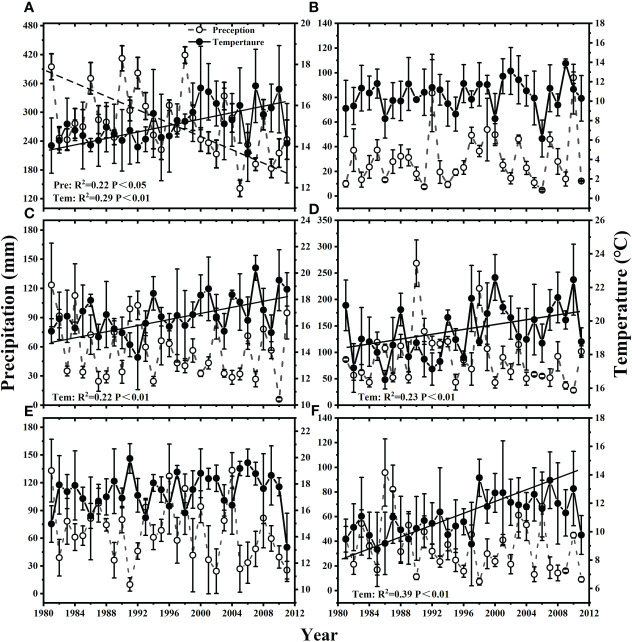
Changes of mean precipitation and mean temperature from 1981 to 2011 during **(A)** the growing season (GS), and the month of **(B)** May, **(C)** June, **(D)** July, **(E)** August and **(F)** September.

Between 1981 and 2011, mean precipitation during the growing season decreased through time at a rate of 3.19 mm year^-1^ (**Figure 1A**). In contrast, mean temperature increased at a rate of 0.08 °C year^-1^ (**Figure 1A**). Mean temperature also increased through time during each of the month of June (0.08°C year^-1^), July (0.09°C year^-1^) and September (0.05°C year^-1^) (**Figures 1C-F**), but mean precipitation did not show any trend through time for any of the month during the growing season.

**Figure 2:**
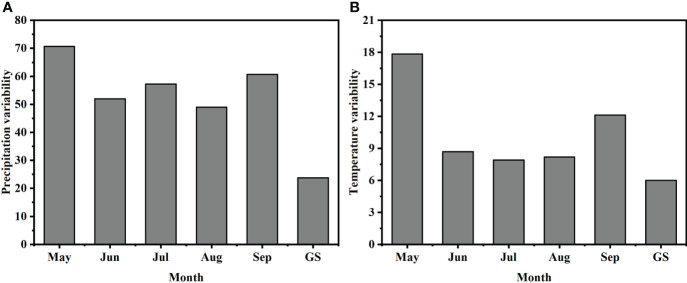
Coefficients of variation of monthly mean **(A)** precipitation and **(B)** temperature over 1981–2011. GS = growing season.

Both precipitation variability and temperature variability between 1981 and 2011 were highest in May, with the lowest variability in precipitation in August and the lowest variability in temperature in July. The variability of precipitation and temperature throughout the growing season was lower than the variability of each month (**Figure 2**).

### Community biomass, species richness, asynchrony and temporal stability

**Figure 3:**
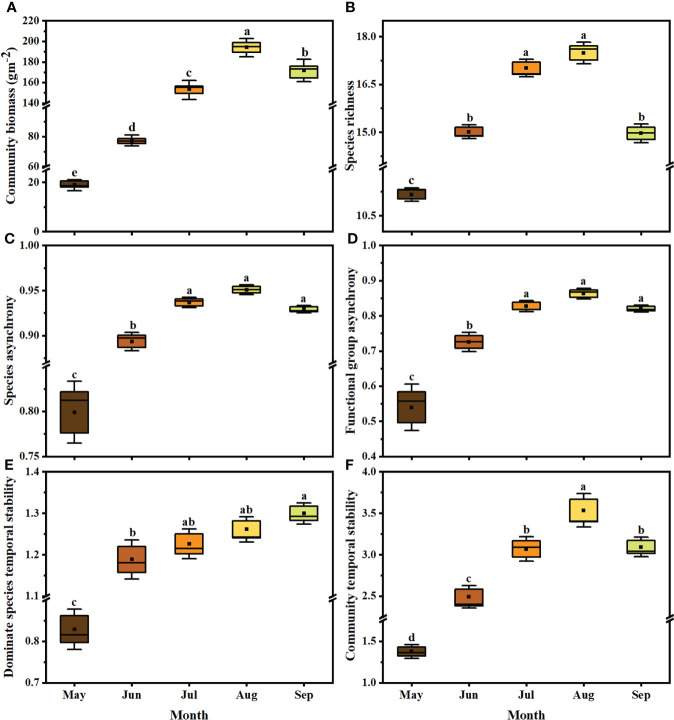
Community biomass **(A)**, species richness **(B)**, species asynchrony **(C)**, functional group asynchrony **(D)**, dominant species temporal stability **(E)** and community temporal stability **(F)** during growing season between 1981 and 2011. Different colors represent the months from May to September (n = 10 each month). The significance of each factor in different months was tested by one-way ANOVA. In Tukey’s HSD’s multi-range test, the different letters at the top of each box represent significant differences between different months (P< 0.05). The boxes indicate the 25-75% confidence interval of each target variable. Solid lines and black squares inside the box represent the median and mean, respectively.

Community biomass (**Figure 3A**), species richness (**Figure 3B**), species asynchrony (**Figure 3C**), functional group asynchrony (**Figure 3D**) and community temporal stability (**Figure 3F**) were highest in August, while dominant species stability was highest in September (**Figure 3E**). During the survey period, community biomass increased in May and June, and decreased from July to September, with a significant trend in August (loss rate 2.61g year^-1^; **Supplementary Figure 3** (a); F_1,28 =_ 28.3, P=0.022; R^2 =^ 0.18) and September (loss rate 3.97g year^-1^; **Supplementary Figure 3** (a); F_1,24 =_ 24.9, P=0.003; R^2 =^ 0.34). Species richness decreased significantly during the growing season from May to September, with loss rates of 2.9, 3.0, 2.4, 2.7, and 2.6 species per decade, respectively (**Supplementary Figure 3** (b); all P<0.05).

### The relationship between community temporal stability and its influencing factors

**Figure 4:**
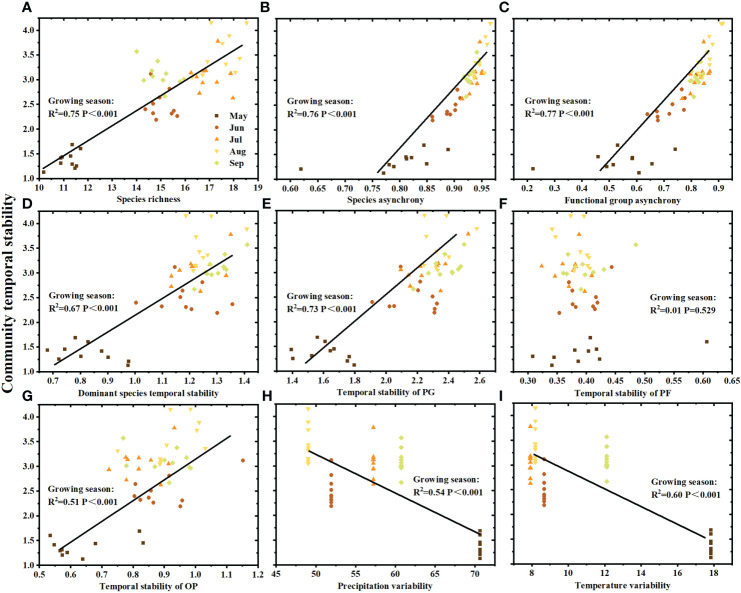
Community temporal stability in relation to **(A)** species richness, **(B)** species asynchrony, **(C)** functional group asynchrony, **(D)** dominant species temporal stability, **(E)** temporal stability of PG (perennial grass), **(F)** temporal stability of PF (perennial forbs), **(G)** temporal stability of OP (other plants), **(H)** precipitation variability and **(I)** temperature variability. Different colors represent the months from May to September (n = 10 each month).

The effects of climatic and biotic factors on community temporal stability showed a seasonal variation. Species richness (**Figure 4A**), species asynchrony (**Figure 4B**), functional group asynchrony (**Figure 4C**), dominant species temporal stability (**Figure 4D**), temporal stability of PG (**Figure 4E**), and temporal stability of OP (**Figure 4G**) throughout the growing season were significantly positively related to community temporal stability, with the exception of temporal stability of PF (**Figure 4F**). Community temporal stability and species asynchrony were significantly positively correlated in May (R^2 =^ 0.42, P=0.042), June (R^2 =^ 0.72, P=0.002), July (R^2 =^ 0.47, P=0.029), August (R^2 =^ 0.76, P=0.042) and September (R^2 =^ 0.64, P=0.005) of the growing season (**Figure 4B**). The significant positive correlation between community temporal stability and functional group asynchrony was observed in June (R^2 =^ 0.68, P=0.003), August (R^2 =^ 0.80, P<0.001) and September (R^2 =^ 0.44, P=0.036) of the growing season (**Figure 4C**). In addition, community temporal stability was positively correlated with dominant species temporal stability (**Figure 4D**, R^2 =^ 0.77, P=0.001) and temporal stability of functional group PG (**Figure 4E**, R^2 =^ 0.42, P=0.039) in September, respectively. Moreover, the variability of total precipitation (**Figure 4H**) and mean temperature (**Figure 4I**) was negatively correlated with community temporal stability.

**Figure 5:**
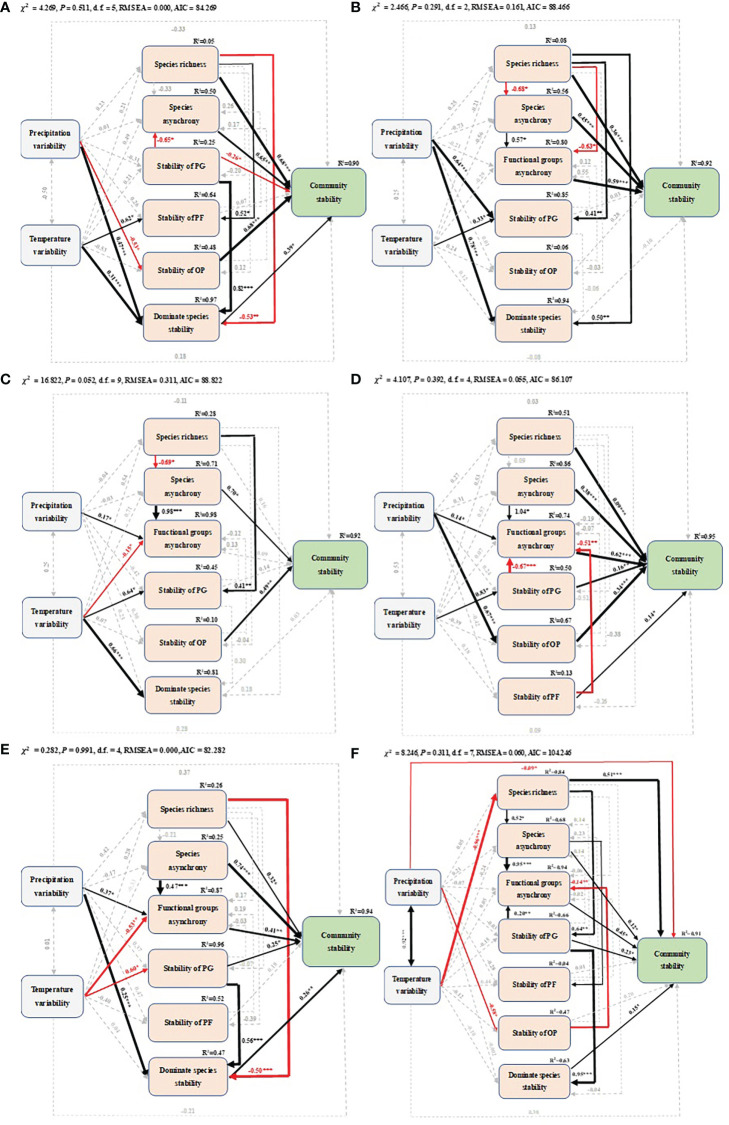
Structural equation models of precipitation (temperature) variability, species richness, species asynchrony, functional group asynchrony, temporal stability of PG (perennial grass), temporal stability of PF (perennial forbs), temporal stability of OP (other plants) and dominant species temporal stability on community temporal stability in different months of the growing season: **(A)** May, **(B)** June, **(C)** July, **(D)** August, **(E)** September and **(F)** the whole growing season. Black and red arrows represent significant positive and negative pathways, respectively, and grey dashed arrows indicate nonsignificant pathways. Arrow width is proportional to the strength of the relationship. Numbers adjacent to arrows are standardized path coefficients and indicate the effect size of the relationship. The proportion of variance explained (R^2^) appears alongside response variables in the model, and asterisks indicate statistical significance (*P< 0.05, **P< 0.01, ***P< 0.001).

SEM showed that climatic factors indirectly influenced community temporal stability throughout the growing season by acting on biotic factors. Species richness, species asynchrony, temporal stability of OP and dominant species temporal stability had direct positive effects on community temporal stability in May. Species richness was positively related to temporal stability of PF, but negatively related to the dominant species temporal stability. Temporal stability of PG indirectly altered community temporal stability by reducing species asynchrony and increasing dominant species temporal stability. Climate variability indirectly affected community temporal stability by increasing temporal stability of PF, dominant species temporal stability and decreasing temporal stability of OP (**Figure 5A**). In June, community temporal stability was positively related to species richness, species asynchrony, and functional group asynchrony. In addition, species richness was positively related to temporal stability of PG, dominant species temporal stability, and negatively related to species asynchrony and functional group asynchrony. Climate variability had a direct positive relationship with temporal stability of PG and dominant species, respectively (**Figure 5B**). In July, species asynchrony and temporal stability of OP had a direct positive relationship with community temporal stability. Species richness had negative and positive relationships with species asynchrony and temporal stability of PG, respectively. Functional group asynchrony was positively related with species asynchrony and precipitation variability and negatively related with temperature variability. Temperature variability also indirectly affected community temporal stability through species asynchrony, temporal stability of PG and dominant species (**Figure 5C**). Specifically, species richness, species and functional group asynchrony, temporal stability of PG, PF and OP were positively related to community temporal stability during peak productivity in August. Functional group asynchrony was positively related to species asynchrony and negatively related to temporal stability of PG and PF. Climate variability indirectly increased community temporal stability by increasing species asynchrony, temporal stability of PG and OP (**Figure 5D**). In September, at the end of the growing season, community temporal stability was positively related to species richness, species and functional group asynchrony, temporal stability of PG, and dominant species temporal stability. The dominant species temporal stability was negatively related to species richness and positively related to temporal stability of PG, respectively. Precipitation variability indirectly increased community temporal stability by increasing functional group asynchrony and dominant species temporal stability. Temperature variability indirectly reduced community temporal stability by decreasing functional group asynchrony and temporal stability of PG (**Figure 5E**). For the whole growing season (May to September), species richness, species asynchrony, functional group asynchrony, dominant species temporal stability and temporal stability of PG were positively related to community temporal stability. In addition, species richness was positively related to species asynchrony and temporal stability of PG. Functional group asynchrony was positively related to species asynchrony and temporal stability of PG, and negatively related to temporal stability of OP. The temporal stability of PG positively related to dominant species temporal stability. Temperature variability and precipitation variability indirectly reduced community temporal stability by reducing species richness and temporal stability of OP. In addition, precipitation variability had a direct negative relationship with community temporal stability (**Figure 5F**).

## Discussion

Our study based on 29 years of field observation revealed lower community temporal stability with higher seasonal climate variability (temperature and precipitation) linked with lower species richness. In addition, we found that species asynchrony, functional group asynchrony and dominant species temporal stability consistently affected the seasonal community temporal stability throughout the growing season.

Climate changes including increased variability in precipitation or temperature, and changed distribution patterns of temperature or precipitation have had an important impact on plant productivity, community composition and species dynamics of the Inner Mongolia temperate grassland over the past several decades (Ma et al., 2010; Zhang et al., 2020). There is growing concern that these changes may in turn affect the temporal stability of community productivity. Previous studies have demonstrated the impact of interannual changes in climate on plant diversity and stability (Zhang et al., 2018; Gilbert et al., 2020). However, the impacts of climate change are likely to be stronger on seasonal scales than on annual scales. Consistent with previous studies, our data showed the continued increase in temperature and decrease in precipitation over the last three decades in the temperate grassland of Inner Mongolia (**Figure 1**; Ma et al., 2010; Li et al., 2015). In addition, we found that for the whole growing season, temperature variability indirectly related to community temporal stability by decreasing species richness (**Figure 5F**). Mechanisms supporting this finding have suggested that significant temperature variability may lead to severe limitations on the physical environment for plant reproduction and high mortality rates of physiological failures such as seedling establishment failure, reduced species richness, and thus weakened community temporal stability (Zhang et al., 2018; Wang et al., 2020; Zhang et al., 2022). In addition, we found that increased temperature variability had a negative effect on functional group asynchrony, but not on species asynchrony, during a period of vigorous plant growth (**Figures 5C–E**). Increased temperature variability reduced the strength of compensation dynamics between functional groups rather than species, possibly because of higher biomass of functional groups relative to species and the increased synchrony of biomass changes between functional groups (Huang et al., 2020). Previous studies have shown that species asynchrony is negatively affected by temperature variability (Ma et al., 2017; Gilbert et al., 2020). However, after considering the effects of species asynchrony and functional group asynchrony on community temporal stability, we found that functional group asynchrony had a greater response to climate variability, possibly because species asynchrony could not fully capture the compensatory dynamics between functional group in our study area (Bai et al., 2004; Zhou et al., 2019). Temperature variability did not directly affect community temporal stability, which might be due to the asymmetry of the effects of daytime and nighttime warming on community temporal stability. Studies have shown that nocturnal warming enhanced community temporal stability, while daytime warming negatively affected community temporal stability, and the two counteracted the significant effects of warming on community temporal stability (Yang et al., 2017).

In contrast to temperature variability, precipitation variability directly reduced community temporal stability during the whole growing season, which is consistent with previous study (**Figure 5F**; Zhang et al., 2018). Precipitation variability had a positive effect on the dominant species temporal stability in May and June at the beginning of the growing season and in September at the end of the growing season. This is due to the fact that the dominant species in the study area are perennial grasses such as *A. cristatum*, *S. grandis* and *L. chinensis*, which are major contributors to community productivity at the beginning and end of the growing season (Loreau and De Mazancourt, 2013; Zhang et al., 2018). Finally, precipitation variability had a positive effect on asynchrony (mainly in functional groups), especially during periods of vigorous plant growth (**Figures 5C–E**), which is consistent with previous studies in the same region (Xu et al., 2015; Chi et al., 2019). Forbs, annual and biennial plants mainly reproduce and grow during this period, and the extreme precipitation events caused by precipitation variability promote the asynchronous response between species (Zhang et al., 2020).

Several field observations and theoretical models have suggested that the stability of biomass may increase with increasing plant diversity (Jiang et al., 2009; Mougi and Kondoh, 2012; Gross et al., 2014; Yan et al., 2021). Here, we found that species richness had a significantly positive relationship with community temporal stability consistently throughout the growing season. Species richness decreased with warming, but increased with increasing precipitation, consistent with previous studies of climate manipulation (Wang and Loreau, 2016; Ma et al., 2017). It is noteworthy that SEM results showed that there was no significant relationship between species richness and community temporal stability in July (**Figure 5C**). This phenomenon might be attributed to rare species (*Axyria amaranthoides*, *Iris tenuifolia* and *Allium tenuissimum)*, generally the most diverse components of a community, limiting community temporal stability through their low abundance (Wang et al., 2020). The seasonal variation of species richness in natural communities showed that diversity-dependent community temporal stability was positively correlated with compensatory effects and asynchronous dynamics among species during the growing season (Bai et al., 2004; Zhang et al., 2018).

Species asynchrony is considered to be a major driver of community temporal stability in the face of climate change (Gross et al., 2014; Craven et al., 2018; Valencia et al., 2020). The asynchrony of population dynamics between species is a common feature of ecological communities (Gonzalez and Loreau, 2009; Blüthgen et al., 2016), and could depend on asynchronous species responses to environmental fluctuations (Ives and Carpenter, 2007; Loreau and De Mazancourt, 2008). Similarly, our study showed positive effects of species asynchrony on community temporal stability throughout the growing season (**Figure 5**). Compensation dynamics among different functional groups is also considered to be an important mechanism of community temporal stability (Bai et al., 2004). We also found that the relationship between functional group asynchrony and community temporal stability remained positive throughout the growing season under climate change. There was also a positive correlation between species asynchrony and functional group. Species asynchrony enhanced community compensation dynamics by promoting functional group asynchrony. Water is the main limiting factor of productivity in arid and semi-arid grassland (Bai et al., 2004; Sala et al., 2012), it creates intense competition among species for water sources (Yang et al., 2011). In the study area observed here, the perennial grasses *L. chinensis* and *A. cristatum* and the perennial forbs *Potentilla bifurca* and *P. tanacetifolia* contributes to community biomass in the early growing season, while the perennial forbs *Axyria amaranthoides*, *Iris tenuifolia* and *Allium tenuissimum* as well as rare annuals and biennials *Orostachys fimbriatus* and *Dysphania aristata* with high growth rate mainly take advantage of precipitation in the late growing season, and are very sensitive to climate variability (**Supplementary Table 7**; Bai et al., 2004; Li et al., 2015; Zhang et al., 2020).

There is growing evidence showing that community temporal stability is positively correlated with dominant species temporal stability (Sasaki and Lauenroth, 2011; Wilsey et al., 2014; Xu et al., 2015). Ecosystems are largely controlled by the characteristics of dominant species, i.e., the mass ratio hypothesis (Grime, 1998), which may even constrain the effect of species diversity on biomass stability (Wayne et al., 2007). The present study also found that the community temporal stability was influenced by the dominant species, reinforcing these ideas. In our study area, the dominant species, two perennial rhizome grasses, *L. chinensis* and *A. cristatum*, and two perennial bunchgrasses, *S. grandis* and *A. sibiricum*, accounted for 62.9% of community above-ground biomass. During the long-term adaptation to climatic conditions and soil nutrients, they have acquired a series of physiological and ecological adaptation characteristics in terms of water use and nutrient acquisition, such as a well-developed root system, high light acquisition capability through a higher canopy, more branches and a larger specific leaf area (Yang et al., 2011; Zhang et al., 2020). These characteristics could make them occupy a dominant position in the community for a long time, so that their stability significantly affected the temporal stability of the community. As the dominant functional group, perennial grasses were not affected by climate variability, and were an important factor maintaining community temporal stability. Because perennial grasses generally have deeper roots, giving them access to deep soil water resources, perennial grasses may have a competitive advantage over forbs and annual herbs in dry conditions (Zhang et al., 2015). It is noteworthy that the effect of climate variability on the temporal stability of dominant species has obvious seasonal dynamics. As described above, the temporal stability of the dominant species is susceptible to climate variability at the beginning or end of the growing season, however during the whole growing season, climate variability had no significant effect on the temporal stability of dominant species.

## Conclusions

Our study provides a new practical basis for climate variability to significantly affect seasonal community temporal stability by affecting species richness, suggesting that increased climate variability has a negative impact on ecosystem functioning. In addition, community temporal stability was mainly affected by species asynchrony, functional group asynchrony and dominant species temporal stability. Under climate change, increased functional group asynchrony could contribute more to community compensatory dynamics and maintain community temporal stability. Based on a long-term study spanning 29 years, our findings elucidate the potential mechanism underlying seasonal climate change on seasonal community temporal stability of temperate grassland in Inner Mongolia, China. Although our study is single-site, our seasonal results are likely to be generalized to other grasslands. This is because many studies assessing multi-sies inter-annual stability found similar contribution of grassland diversity, species stability and asynchrony, as well as functional group stability and asynchrony to temporal stability (Valencia et al., 2020; Muraina et al., 2021; Schnabel et al., 2021). Similarly, given the increasing evidence that these mechanisms act at multiple spatial scales (Hautier et al., 2020) and in other ecosystems (Xu et al., 2021), our results should hold at larger spatial scales and among ecosystems. However, future coordinated multi-sites, larger-scale studies in other ecosystems than grasslands are needed to test whether our seasonal results are universal.

## Data availability statement

The original contributions presented in the study are included in the article/supplementary files. Further inquiries can be directed to Ze Zhang, zzhang0514@126.com.

## Author contributions

ZZ, YH, HQ and ZL raised scientific questions and designed the experiments. TB, JY and ZL conducted the experiments. ZZ, MW, TL, MY and GZ analyzed the experimental data. ZZ, YH and HQ wrote the manuscript. All authors contributed to the article and approved the submitted version.

## Funding

This study was financially supported by the Major Science and Technology Projects of Inner Mongolia Autonomous Region (2020ZD0009), the Applied Technology Research and Development Projects of Inner Mongolia Autonomous Region (2019GG015), and the Natural Science Foundation of Inner Mongolia Autonomous Region (2022MS03036, 2019BS03008).

## Conflict of interest

The authors declare that the research was conducted in the absence of any commercial or financial relationships that could be construed as a potential conflict of interest.

## Publisher’s note

All claims expressed in this article are solely those of the authors and do not necessarily represent those of their affiliated organizations, or those of the publisher, the editors and the reviewers. Any product that may be evaluated in this article, or claim that may be made by its manufacturer, is not guaranteed or endorsed by the publisher.
