# Supplementary material for: Species richness and asynchrony maintain the stability of primary productivity against seasonal climatic variability
Source: Front Plant Sci. 2022 Oct 28;13:1014049. doi: 10.3389/fpls.2022.1014049 (PMC9650401; doi:10.3389/fpls.2022.1014049)
Supplement: Supplementary file 1 [file DataSheet_1.docx]

**Supporting information**


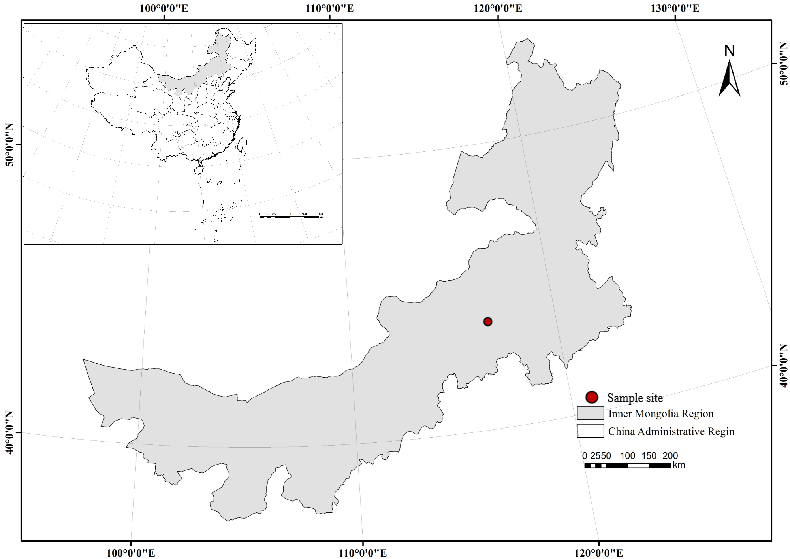


**Figure S1.** Location of sampling area (IMGRS) of study region in Inner Mongolia of China.


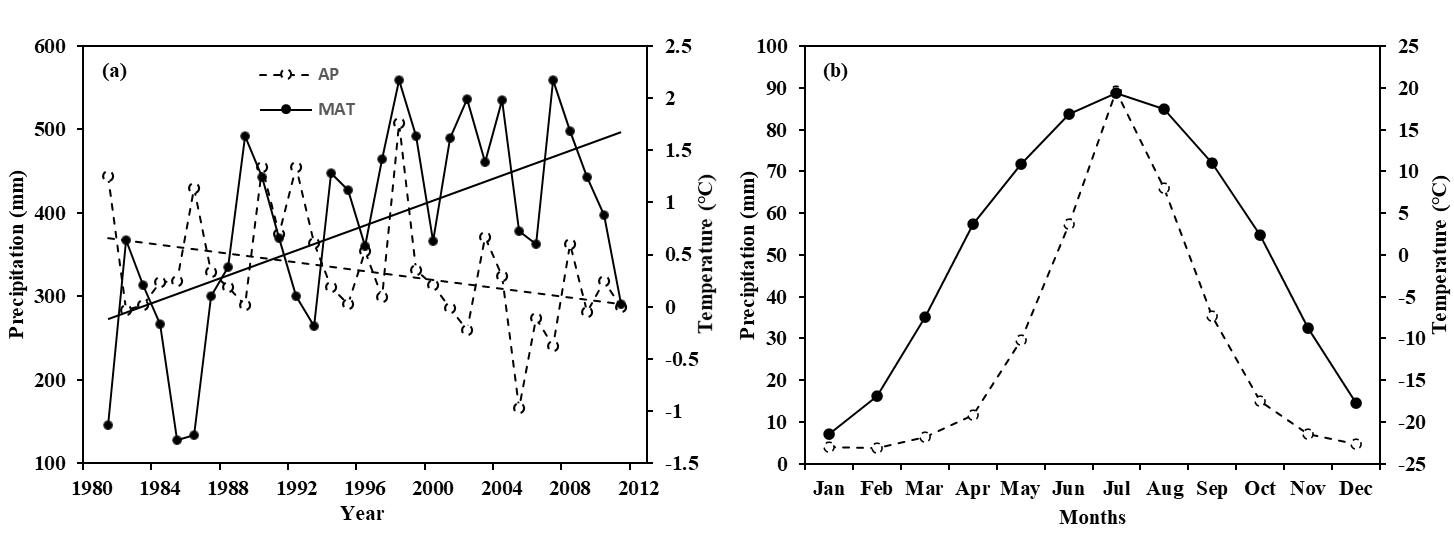


**Figure S2.** During the period 1981 to 2011, (a) interannual variation of mean annual temperature and annual precipitation and (b) mean temperature and precipitation for different months at the study site. Annual precipitation (AP), Mean annual temperature (MAT).


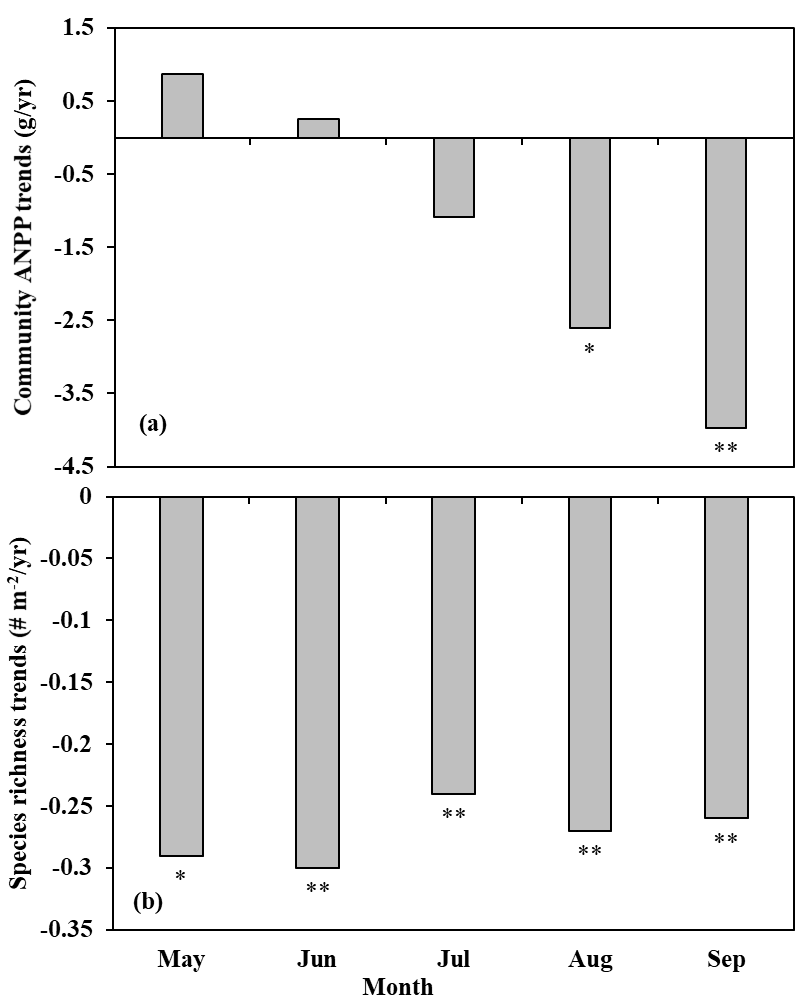


**Figure S3.** Trends of (a) community biomass and (b) species richness in growing seasons from 1981 to 2011.

**Table S1.** Results of structural equation modeling of precipitation variability and temperature variability effects on plant community temporal stability through all plausible interaction pathways in **May**. Given are the standardized path coefficients (estimates), standard error of regression weight (S.E.), the critical value for the regression weight (C.R.), and the level of significance for the regression weight (P). *** indicates P < 0.001.

|  |  | Path | Estimate | S.E. | C.R. | P |
| --- | --- | --- | --- | --- | --- | --- |
| Temperature variability | <--- | Precipitation variability | -.500 | .289 | -1.732 | .083 |
| Species richness | <--- | Temperature variability | 1.963 | 3.558 | .552 | .581 |
| Species richness | <--- | Precipitation variability | 2.157 | 3.558 | .606 | .544 |
| Temporal stability of PG | <--- | Precipitation variability | -.936 | 1.026 | -.913 | .361 |
| Temporal stability of PG | <--- | Temperature variability | .628 | 1.023 | .614 | .539 |
| Temporal stability of OP | <--- | Temperature variability | -1.234 | .655 | -1.884 | .060 |
| Temporal stability of OP | <--- | Precipitation variability | -1.866 | .657 | -2.840 | .005 |
| Temporal stability of PF | <--- | Precipitation variability | .445 | .403 | 1.103 | .270 |
| Temporal stability of PF | <--- | Temperature variability | 1.060 | .402 | 2.636 | .008 |
| Temporal stability of PF | <--- | Species richness | .094 | .037 | 2.524 | .012 |
| Temporal stability of PG | <--- | Species richness | -.064 | .094 | -.674 | .500 |
| Temporal stability of OP | <--- | Species richness | .029 | .060 | .482 | .630 |
| Species asynchrony | <--- | Precipitation variability | .013 | .676 | .020 | .984 |
| Species asynchrony | <--- | Temperature variability | .714 | .655 | 1.090 | .276 |
| Species asynchrony | <--- | Temporal stability of PF | .215 | .352 | .609 | .543 |
| Species asynchrony | <--- | Temporal stability of PG | -.322 | .144 | -2.226 | .026 |
| Species asynchrony | <--- | Temporal stability of OP | .109 | .230 | .476 | .634 |
| Species asynchrony | <--- | Species richness | -.051 | .049 | -1.044 | .296 |
| Dominate species temporal stability | <--- | Temperature variability | .664 | .182 | 3.644 | *** |
| Dominate species temporal stability | <--- | Precipitation variability | 1.009 | .158 | 6.391 | *** |
| Dominate species temporal stability | <--- | Species richness | -.044 | .015 | -2.999 | .003 |
| Dominate species temporal stability | <--- | Temporal stability of PG | .582 | .065 | 8.893 | *** |
| Dominate species temporal stability | <--- | Species asynchrony | -.385 | .119 | -3.248 | .001 |
| Community temporal stability | <--- | Temporal stability of PG | -1.436 | .636 | -2.259 | .024 |
| Community temporal stability | <--- | Temporal stability of PF | .134 | .402 | .333 | .739 |
| Community temporal stability | <--- | Temporal stability of OP | 1.042 | .258 | 4.038 | *** |
| Community temporal stability | <--- | Species asynchrony | 1.590 | .507 | 3.137 | .002 |
| Community temporal stability | <--- | Species richness | .223 | .064 | 3.463 | *** |
| Community temporal stability | <--- | Dominate species temporal stability | 2.219 | 1.061 | 2.092 | .036 |
| Community temporal stability | <--- | Precipitation variability | -1.159 | 1.208 | -.960 | .337 |
| Community temporal stability | <--- | Temperature variability | .646 | 1.035 | .624 | .533 |

**Table S2.** Results of structural equation modeling of precipitation variability and temperature variability effects on plant community temporal stability through all plausible interaction pathways in **June**. Given are the standardized path coefficients (estimates), standard error of regression weight (S.E.), the critical value for the regression weight (C.R.), and the level of significance for the regression weight (P). *** indicates P < 0.001.

|  |  | Path | Estimate | S.E. | C.R. | P |
| --- | --- | --- | --- | --- | --- | --- |
| Temperature variability | <--- | Precipitation variability | .250 | .323 | .775 | .439 |
| Species richness | <--- | Precipitation variability | 172.334 | 228.084 | .756 | .450 |
| Species richness | <--- | Temperature variability | -145.333 | 228.084 | -.637 | .524 |
| Temporal stability of PG | <--- | Precipitation variability | 139.674 | 30.222 | 4.622 | *** |
| Temporal stability of PG | <--- | Temperature variability | 71.776 | 29.961 | 2.396 | .017 |
| Temporal stability of OP | <--- | Precipitation variability | 1.814 | 54.036 | .034 | .973 |
| Temporal stability of OP | <--- | Temperature variability | 37.366 | 53.568 | .698 | .485 |
| Temporal stability of PG | <--- | Species richness | .131 | .043 | 3.070 | .002 |
| Temporal stability of OP | <--- | Species richness | -.007 | .077 | -.087 | .931 |
| Species asynchrony | <--- | Precipitation variability | -24.449 | 15.409 | -1.587 | .113 |
| Species asynchrony | <--- | Temperature variability | -18.825 | 11.024 | -1.708 | .088 |
| Species asynchrony | <--- | Species richness | -.033 | .017 | -1.992 | .046 |
| Species asynchrony | <--- | Temporal stability of PG | .118 | .094 | 1.245 | .213 |
| Species asynchrony | <--- | Temporal stability of OP | .100 | .053 | 1.886 | .059 |
| Functional group asynchrony | <--- | Precipitation variability | -19.202 | 31.756 | -.605 | .545 |
| Functional group asynchrony | <--- | Temperature variability | -26.913 | 23.108 | -1.165 | .244 |
| Functional group asynchrony | <--- | Species asynchrony | 1.539 | .607 | 2.535 | .011 |
| Functional group asynchrony | <--- | Species richness | -.083 | .036 | -2.284 | .022 |
| Functional group asynchrony | <--- | Temporal stability of PG | .229 | .186 | 1.230 | .219 |
| Functional group asynchrony | <--- | Temporal stability of OP | .071 | .114 | .627 | .531 |
| Dominate species temporal stability | <--- | Precipitation variability | 116.566 | 25.561 | 4.560 | *** |
| Dominate species temporal stability | <--- | Temperature variability | 18.413 | 18.436 | .999 | .318 |
| Dominate species temporal stability | <--- | Species richness | .109 | .037 | 2.982 | .003 |
| Dominate species temporal stability | <--- | Temporal stability of PG | -.041 | .157 | -.260 | .795 |
| Dominate species temporal stability | <--- | Species asynchrony | 1.726 | .708 | 2.437 | .015 |
| Dominate species temporal stability | <--- | Functional group asynchrony | -.586 | .302 | -1.942 | .052 |
| Community temporal stability | <--- | Species asynchrony | 6.446 | .655 | 9.848 | *** |
| Community temporal stability | <--- | Species richness | .255 | .038 | 6.761 | *** |
| Community temporal stability | <--- | Dominate species temporal stability | -.329 | .253 | -1.300 | .194 |
| Community temporal stability | <--- | Temporal stability of PG | .056 | .134 | .417 | .677 |
| Community temporal stability | <--- | Temporal stability of OP | .863 | .081 | 10.599 | *** |
| Community temporal stability | <--- | Functional group asynchrony | 3.093 | .275 | 11.246 | *** |
| Community temporal stability | <--- | Precipitation variability | 60.972 | 33.428 | 1.824 | .068 |
| Community temporal stability | <--- | Temperature variability | 37.433 | 16.483 | 2.271 | .023 |

**Table S3.** Results of structural equation modeling of precipitation variability and temperature variability effects on plant community temporal stability through all plausible interaction pathways in **July**. Given are the standardized path coefficients (estimates), standard error of regression weight (S.E.), the critical value for the regression weight (C.R.), and the level of significance for the regression weight (P). *** indicates P < 0.001.

|  |  | Path | Estimate | S.E. | C.R. | P |
| --- | --- | --- | --- | --- | --- | --- |
| Temperature variability | <--- | Precipitation variability | .250 | .323 | .775 | .439 |
| Species richness | <--- | Precipitation variability | -29.200 | 219.499 | -.133 | .894 |
| Species richness | <--- | Temperature variability | 403.800 | 219.498 | 1.840 | .066 |
| Species asynchrony | <--- | Precipitation variability | -.430 | 3.090 | -.139 | .889 |
| Species asynchrony | <--- | Temperature variability | 11.916 | 3.621 | 3.291 | .001 |
| Temporal stability of PG | <--- | Precipitation variability | -81.334 | 49.902 | -1.630 | .103 |
| Temporal stability of PG | <--- | Temperature variability | 125.333 | 49.902 | 2.512 | .012 |
| Temporal stability of OP | <--- | Temperature variability | 6.733 | 32.718 | .206 | .837 |
| Temporal stability of OP | <--- | Precipitation variability | 30.067 | 32.718 | .919 | .358 |
| Species asynchrony | <--- | Species richness | -.022 | .005 | -4.604 | *** |
| Functional group asynchrony | <--- | Precipitation variability | 7.353 | 3.019 | 2.436 | .015 |
| Functional group asynchrony | <--- | Temperature variability | -6.584 | 2.977 | -2.212 | .027 |
| Functional group asynchrony | <--- | Species asynchrony | 2.604 | .150 | 17.303 | *** |
| Functional group asynchrony | <--- | Temporal stability of PG | .029 | .018 | 1.582 | .114 |
| Functional group asynchrony | <--- | Temporal stability of OP | -.051 | .028 | -1.828 | .068 |
| Dominate specie temporal stability | <--- | Precipitation variability | 26.300 | 20.082 | 1.310 | .190 |
| Dominate specie temporal stability | <--- | Temperature variability | 75.187 | 22.613 | 3.325 | *** |
| Dominate specie temporal stability | <--- | Temporal stability of PG | .172 | .120 | 1.435 | .151 |
| Dominate species temporal stability | <--- | Functional group asynchrony | -.474 | .379 | -1.250 | .211 |
| Community temporal stability | <--- | Species asynchrony | 21.155 | 15.958 | 1.326 | .185 |
| Community temporal stability | <--- | Temperature variability | 143.253 | 158.781 | .902 | .367 |
| Community temporal stability | <--- | Precipitation variability | -54.521 | 65.054 | -.838 | .402 |
| Community temporal stability | <--- | Dominate species temporal stability | .132 | 1.110 | .119 | .905 |
| Community temporal stability | <--- | Temporal Stability of PG | .370 | .378 | .980 | .327 |
| Community temporal stability | <--- | Temporal stability of OP | 2.455 | .813 | 3.020 | .003 |
| Community temporal stability | <--- | Functional group asynchrony | -.998 | 5.603 | -.178 | .859 |
| Community temporal stability | <--- | Species richness | -.106 | .155 | -.685 | .493 |

**Table S4.** Results of structural equation modeling of precipitation variability and temperature variability effects on plant community temporal stability through all plausible interaction pathways in **August**. Given are the standardized path coefficients (estimates), standard error of regression weight (S.E.), the critical value for the regression weight (C.R.), and the level of significance for the regression weight (P). *** indicates P < 0.001.

|  |  | Path | Estimate | S.E. | C.R. | P |
| --- | --- | --- | --- | --- | --- | --- |
| Temperature variability | <--- | Precipitation variability | .050 | .027 | 1.861 | .063 |
| Species richness | <--- | Precipitation variability | 29.504 | 29.831 | .989 | .323 |
| Species richness | <--- | Temperature variability | 605.923 | 314.450 | 1.927 | .054 |
| Temporal stability of PG | <--- | Precipitation variability | 3.521 | 4.382 | .804 | .422 |
| Temporal stability of PG | <--- | Temperature variability | 130.504 | 52.132 | 2.503 | .012 |
| Temporal stability of OP | <--- | Precipitation variability | 12.628 | 2.990 | 4.223 | *** |
| Temporal stability of OP | <--- | Temperature variability | -51.817 | 35.580 | -1.456 | .145 |
| Temporal stability of PF | <--- | Precipitation variability | -1.481 | 1.301 | -1.139 | .255 |
| Temporal stability of PF | <--- | Temperature variability | 6.615 | 13.710 | .483 | .629 |
| Temporal stability of PG | <--- | Species richness | -.071 | .046 | -1.518 | .129 |
| Temporal stability of OP | <--- | Species richness | -.044 | .032 | -1.390 | .165 |
| Species asynchrony | <--- | Precipitation variability | .591 | .545 | 1.084 | .278 |
| Species asynchrony | <--- | Temperature variability | 11.524 | 4.952 | 2.327 | .020 |
| Species asynchrony | <--- | Species richness | .002 | .004 | .411 | .681 |
| Species asynchrony | <--- | Temporal stability of PG | -.086 | .024 | -3.567 | *** |
| Species asynchrony | <--- | Temporal stability of OP | -.011 | .040 | -.280 | .779 |
| Species asynchrony | <--- | Temporal stability of PF | -.278 | .092 | -3.026 | .002 |
| Functional group asynchrony | <--- | Precipitation variability | -.743 | 2.224 | -.334 | .738 |
| Functional group asynchrony | <--- | Temperature variability | 3.959 | 24.036 | .165 | .869 |
| Functional group asynchrony | <--- | Species asynchrony | 2.930 | 1.278 | 2.292 | .022 |
| Functional group asynchrony | <--- | Species richness | -.010 | .015 | -.632 | .527 |
| Functional group asynchrony | <--- | Temporal stability of PF | .060 | .500 | .120 | .905 |
| Functional group asynchrony | <--- | Temporal stability of PG | .046 | .144 | .319 | .749 |
| Functional group asynchrony | <--- | Temporal stability of OP | -.034 | .154 | -.223 | .823 |
| Community temporal stability | <--- | Species asynchrony | 12.538 | .187 | 66.943 | *** |
| Community temporal stability | <--- | Species richness | -.054 | .002 | -29.810 | *** |
| Community temporal stability | <--- | Temporal stability of PG | .669 | .017 | 39.749 | *** |
| Community temporal stability | <--- | Temporal stability of OP | 1.718 | .018 | 95.711 | *** |
| Community temporal stability | <--- | Temporal stability of PF | 2.476 | .058 | 42.510 | *** |
| Community temporal stability | <--- | Functional group asynchrony | 7.811 | .039 | 201.285 | *** |
| Community temporal stability | <--- | Precipitation variability | 1.976 | .261 | 7.585 | .067 |
| Community temporal stability | <--- | Temperature variability | 64.635 | 2.803 | 23.063 | *** |

**Table S5.** Results of structural equation modeling of precipitation variability and temperature variability effects on plant community temporal stability through all plausible interaction pathways in **September**. Given are the standardized path coefficients (estimates), standard error of regression weight (S.E.), the critical value for the regression weight (C.R.), and the level of significance for the regression weight (P). *** indicates P < 0.001.

|  |  | Path | Estimate | S.E. | C.R. | P |
| --- | --- | --- | --- | --- | --- | --- |
| Temperature variability | <--- | Precipitation variability | .000 | .333 | .000 | 1.000 |
| Species richness | <--- | Precipitation variability | 385.500 | 264.286 | 1.459 | .145 |
| Species richness | <--- | Temperature variability | 260.500 | 264.286 | .986 | .324 |
| Species asynchrony | <--- | Precipitation variability | -2.145 | 4.102 | -.523 | .601 |
| Species asynchrony | <--- | Temperature variability | -4.253 | 3.883 | -1.095 | .273 |
| Species asynchrony | <--- | Species richness | -.003 | .005 | -.616 | .538 |
| Temporal stability of PG | <--- | Precipitation variability | 58.775 | 39.272 | 1.497 | .134 |
| Temporal stability of PG | <--- | Temperature variability | -91.442 | 37.176 | -2.460 | .014 |
| Temporal stability of PF | <--- | Precipitation variability | 26.499 | 15.026 | 1.764 | .078 |
| Temporal stability of PF | <--- | Temperature variability | -22.500 | 14.224 | -1.582 | .114 |
| Temporal stability of PG | <--- | Species richness | -.012 | .045 | -.264 | .792 |
| Temporal stability of PF | <--- | Species richness | -.024 | .017 | -1.408 | .159 |
| Functional group asynchrony | <--- | Precipitation variability | 11.156 | 5.037 | 2.215 | .027 |
| Functional group asynchrony | <--- | Temperature variability | -15.974 | 5.391 | -2.963 | .003 |
| Functional group asynchrony | <--- | Species asynchrony | 1.099 | .324 | 3.389 | *** |
| Functional group asynchrony | <--- | Species richness | .006 | .005 | 1.099 | .272 |
| Functional group asynchrony | <--- | Temporal stability of PF | .104 | .088 | 1.177 | .239 |
| Functional group asynchrony | <--- | Temporal stability of PG | -.005 | .034 | -.156 | .876 |
| Dominate species temporal stability | <--- | Precipitation variability | 19.823 | 3.059 | 6.480 | *** |
| Dominate species temporal stability | <--- | Temperature variability | 3.265 | 3.728 | .876 | .381 |
| Dominate species temporal stability | <--- | Temporal stability of PG | .295 | .017 | 17.764 | *** |
| Dominate species temporal stability | <--- | Species asynchrony | 1.499 | .228 | 6.589 | *** |
| Dominate species temporal stability | <--- | Species richness | -.044 | .002 | -18.897 | *** |
| Dominate species temporal stability | <--- | Functional group asynchrony | .433 | .153 | 2.837 | .005 |
| Community temporal stability | <--- | Species asynchrony | 28.963 | 2.810 | 10.308 | *** |
| Community temporal stability | <--- | Species richness | -.297 | .077 | -3.841 | *** |
| Community temporal stability | <--- | Dominate species temporal stability | -5.508 | 1.721 | -3.201 | .001 |
| Community temporal stability | <--- | Temporal stability of PG | 2.632 | .516 | 5.106 | *** |
| Community temporal stability | <--- | Temporal stability of PF | 1.180 | .241 | 4.897 | *** |
| Community temporal stability | <--- | Functional group asynchrony | -1.282 | 1.150 | -1.114 | .265 |
| Community temporal stability | <--- | Precipitation variability | 133.056 | 37.808 | 3.519 | *** |
| Community temporal stability | <--- | Temperature variability | -75.806 | 19.886 | -3.812 | *** |

**Table S6.** Results of structural equation modeling of precipitation variability and temperature variability effects on plant community temporal stability through all plausible interaction pathways over the whole growing season (**May to** **September)**. Given are the standardized path coefficients (estimates), standard error of regression weight (S.E.), the critical value for the regression weight (C.R.), and the level of significance for the regression weight (P). *** indicates P < 0.001.

|  |  | Path | Estimate | S.E. | C.R. | P |
| --- | --- | --- | --- | --- | --- | --- |
| Temperature variability | <--- | Precipitation variability | .459 | .027 | 16.678 | *** |
| Species richness | <--- | Precipitation variability | .016 | .046 | .351 | .726 |
| Species richness | <--- | Temperature variability | -.588 | .092 | -6.424 | *** |
| Temporal stability of PG | <--- | Precipitation variability | -.001 | .009 | -.156 | .876 |
| Temporal stability of PG | <--- | Temperature variability | -.012 | .024 | -.523 | .601 |
| Temporal stability of OP | <--- | Precipitation variability | -.010 | .005 | -2.169 | .030 |
| Temporal stability of OP | <--- | Temperature variability | -.004 | .010 | -.430 | .667 |
| Temporal stability of PF | <--- | Precipitation variability | -.002 | .002 | -.785 | .432 |
| Temporal stability of PF | <--- | Temperature variability | .005 | .004 | 1.220 | .223 |
| Temporal stability of PG | <--- | Species richness | .085 | .027 | 3.103 | .002 |
| Species asynchrony | <--- | Precipitation variability | .002 | .002 | .963 | .336 |
| Species asynchrony | <--- | Temperature variability | -.004 | .005 | -.849 | .396 |
| Species asynchrony | <--- | Species richness | .014 | .006 | 2.394 | .017 |
| Species asynchrony | <--- | Temporal stability of PG | .029 | .030 | .978 | .328 |
| Species asynchrony | <--- | Temporal stability of OP | .107 | .055 | 1.954 | .051 |
| Species asynchrony | <--- | Temporal stability of PF | .209 | .119 | 1.750 | .080 |
| Functional group asynchrony | <--- | Precipitation variability | -.001 | .002 | -.769 | .442 |
| Functional group asynchrony | <--- | Temperature variability | .002 | .004 | .421 | .674 |
| Functional group asynchrony | <--- | Species asynchrony | 2.009 | .126 | 15.888 | *** |
| Functional group asynchrony | <--- | Species richness | -.004 | .006 | -.673 | .501 |
| Functional group asynchrony | <--- | Temporal stability of PF | -.071 | .109 | -.653 | .514 |
| Functional group asynchrony | <--- | Temporal stability of PG | .087 | .027 | 3.224 | .001 |
| Functional group asynchrony | <--- | Temporal stability of OP | -.141 | .050 | -2.791 | .005 |
| Dominate species temporal stability | <--- | Precipitation variability | .000 | .002 | -.019 | .985 |
| Dominate species temporal stability | <--- | Temperature variability | -.005 | .007 | -.732 | .464 |
| Dominate species temporal stability | <--- | Species richness | -.003 | .009 | -.378 | .705 |
| Dominate species temporal stability | <--- | Temporal stability of PG | .581 | .043 | 13.391 | *** |
| Dominate species temporal stability | <--- | Species asynchrony | -.256 | .444 | -.577 | .564 |
| Dominate species temporal stability | <--- | Functional group asynchrony | .053 | .211 | .249 | .803 |
| Community temporal stability | <--- | Species asynchrony | -.300 | 2.405 | -.125 | .901 |
| Community temporal stability | <--- | Species richness | .173 | .042 | 4.073 | *** |
| Community temporal stability | <--- | Dominate species temporal stability | -.021 | .692 | -.030 | .976 |
| Community temporal stability | <--- | Temporal stability of PG | .596 | .446 | 1.334 | .182 |
| Community temporal stability | <--- | Temporal stability of OP | 1.160 | .418 | 2.779 | .005 |
| Community temporal stability | <--- | Temporal stability of PF | .006 | .829 | .007 | .995 |
| Community temporal stability | <--- | Functional group asynchrony | 2.638 | 1.087 | 2.427 | .015 |
| Community temporal stability | <--- | Precipitation variability | .009 | .013 | .714 | .017 |
| Community temporal stability | <--- | Temperature variability | -.079 | .033 | -2.393 | .475 |

**Table S7.** Common species and their relative abundance during the long-term observation period from 1981 to 2011.

| Species |  | Functional group |  | Relative abundance |  | Species |  | Functional group |  | Relative abundance |
| --- | --- | --- | --- | --- | --- | --- | --- | --- | --- | --- |
| *Leymus chinensis* |  | PR |  | 25.5 ± 4.2 % |  | *Stipa*  *grandis* |  | PB |  | 19.1 ± 3.6 % |
| *Agropyron cristatum* |  | PR |  | 7.1 ± 1.6 % |  | *Achnatherum sibiricum* |  | PB |  | 11.2 ± 2.7 % |
| *Carex korshinskii* |  | PR |  | 2.9 ± 1.2 % |  | *Cleistogenes squarrosa* |  | PB |  | 1.92 ± 1.1 % |
| *Heteropappus altaicus* |  | PF |  | 0.73 ± 0.7 % |  | *Poa*  *attenuata* |  | PB |  | 0.74 ± 0.9 % |
| *Allium anisopodium* |  | PF |  | 0.65 ± 0.6 % |  | *Koeleria*  *macrantha* |  | PB |  | 0.34 ± 0.4 % |
| *Pulsatilla*  *turczaninovi* |  | PF |  | 0.47 ± 0.3 % |  | *Thalictrum petaloideum* |  | PF |  | 0.99 ± 0.7 % |
| *Medicago ruthenica* |  | PF |  | 0.21 ± 0.3 % |  | *Artemisia pubescens* |  | PF |  | 0.28 ± 0.2 % |
| *Phlomis mongolica* |  | PF |  | 0.22 ± 0.4 % |  | *Haplophyllum dauricum* |  | PF |  | 0.31 ± 0.3 % |
| *Oxytropis myriophylla* |  | PF |  | 0.82 ± 1.0 % |  | *Potentilla bifurca* |  | PF |  | 0.94 ± 0.9 % |
| *Limonium bicolor* |  | PF |  | 0.24 ± 0.08 % |  | *Allium condensatum* |  | PF |  | 0.48 ± 0.1 % |
| *Saposhnikovia divaricata* |  | PF |  | 0.28 ± 0.09 % |  | *Potentilla tanacetifolia* |  | PF |  | 0.84 ± 0.5 % |
| *Glycyrrhiza uralensis* |  | PF |  | 0.27 ± 0.04 % |  | *Potentilla verticillaris* |  | PF |  | 0.81 ± 0.4 % |
| *Tephroseris kirilowii* |  | PF |  | 0.30 ± 0.07 % |  | *Klasea centauroides* |  | PF |  | 0.78 ± 0.6 % |
| *Leontopodiu leontopodioides* |  | PF |  | 0.31 ± 0.07 % |  | *Pedicularis striata* |  | PF |  | 0.58 ± 0.2 % |
| *Linariavulgaris subsp. sinensis* |  | PF |  | 0.38 ± 0.04 % |  | *Astragalus galactites* |  | PF |  | 0.62 ± 0.3 % |
| *Galium verum* |  | PF |  | 0.31 ± 0.07 % |  | *Adenophora stenanthina* |  | PF |  | 0.57 ± 0.3 % |
| *Thermopsis lanceolata* |  | PF |  | 0.32 ± 0.04 % |  | *Allium senescens* |  | PF |  | 0.52 ± 0.2 % |
| *Euphorbia esula* |  | PF |  | 0.26 ± 0.05 % |  | *Allium tenuissimum* |  | PF |  | 0.91 ± 0.6 % |
| *Sibbaldia adpressa* |  | PF |  | 0.31 ± 0.06 % |  | *Iris*  *tenuifolia* |  | PF |  | 0.72 ± 0.4 % |
| *Allium*  *bidentatum* |  | PF |  | 0.31 ± 0.02 % |  | *Potentilla acaulis* |  | PF |  | 0.91 ± 0.3 % |
| *Phedimus aizoon* |  | PF |  | 0.29 ± 0.01 % |  | *Linum*  *perenne* |  | PF |  | 0.41 ± 0.1 % |
| *Bupleurum scorzonerifolium* |  | PF |  | 0.20 ± 0.03 % |  | *Allium ramosum* |  | PF |  | 0.52 ± 0.2 % |
| *Gueldenstaedtia verna* |  | PF |  | 0.18 ± 0.02 % |  | *Astragalus adsurgens* |  | PF |  | 0.46 ± 0.2 % |
| *Cymbaria daurica* |  | PF |  | 0.19 ± 0.03 % |  | *Nepeta multifida* |  | PF |  | 0.57 ± 0.2 % |
| *Silene jenisseensis* |  | PF |  | 0.34 ± 0.06 % |  | *Silene*  *aprica* |  | PF |  | 0.38 ± 0.1 % |
| *Saussurea japonica var pteroclada* |  | PF |  | 0.21 ± 0.04 % |  | *Caragana microphylla* |  | SS |  | 3.39 ± 0.9 % |
| *Artemisia frigida* |  | SS |  | 2.41 ± 0.6 % |  | *Kochia prostrata* |  | SS |  | 0.7 ± 0.3 % |
| *Dysphania aristata* |  | AB |  | 0.23 ± 0.2 % |  | *Orostachys fimbriatus* |  | AB |  | 0.3 ± 0.1 % |
| *Chenopodium album* |  | AB |  | 0.67 ± 0.7 % |  | *Dontostemon micranthus* |  | AB |  | 0.68 ± 0.5 % |
| *Gentiana squarrosa* |  | AB |  | 0.42 ± 0.2 % |  | *Axyris amaranthoides* |  | AB |  | 0.61 ± 0.4 % |
| *Salsola collina* |  | AB |  | 1.39 ± 0.4 % |  |  |  |  |  |  |

Abbreviations: perennial rhizome grass (PR), perennial bunchgrasses (PB), perennial forbs (PF), shrubs and semi-shrubs (SS), and annuals and biennials (AB).
